# MicroRNAs-mediated regulation of glucose transporter (GLUT) expression in glioblastoma

**DOI:** 10.1016/j.ncrna.2022.09.001

**Published:** 2022-09-06

**Authors:** Ozal Beylerli, Galina Sufianova, Alina Shumadalova, Daming Zhang, Ilgiz Gareev

**Affiliations:** a Рeoples’ Friendship University of Russia (RUDN University), 6 Miklukho-Maklaya Street, Moscow, 117198, Russian Federation; b Bashkir State Medical University, Ufa, Republic of Bashkortostan, 450008, Russia; c Department of Pharmacology, Tyumen State Medical University, 54 Odesskaya Street, 625023, Tyumen, Russia; d Department of Neurosurgery, The First Affiliated Hospital of Harbin Medical University, Harbin, 150001, China

**Keywords:** Glioblastoma, GLUT proteins, miRNAs, Glucose metabolism, HIF-1α, Angiogenesis

## Abstract

Current knowledge about the role of microRNAs (miRNAs) in tumor glucose metabolism is growing, and a number of studies regularly confirm the impact miRNAs can have on glucose metabolism reprogramming in tumors. However, there remains a lack of understanding of the broader perspective on the role of miRNAs in energy reprogramming in glioblastoma. An important role in the metabolism of glucose is played by carrier proteins that ensure its transmembrane movement. Carrier proteins in mammalian cells are glucose transporters (GLUTs). In total, 12 types of GLUTs are distinguished, differing in localization, affinity for glucose and ability to regulate. The fact of increased consumption of glucose in tumors compared to non-proliferating normal tissues is known. Tumor cells need glucose to ensure their survival and growth, so the type of transport proteins like GLUT are critical for them. Previous studies have shown that GLUT-1 and GLUT-3 may play an important role in the development of some types of malignant tumors, including glioblastoma. In addition, there is evidence of how GLUT-1 and GLUT-3 expression is regulated by miRNAs in glioblastoma. Thus, the aim of this study is to highlight the role of specific miRNAs in modulating GLUT levels in order to take into account the use of miRNAs expression modulators as a useful strategy to increase the sensitivity of glioblastoma to current therapies.

## 1 Introduction

The glucose transporter (GLUT) family of proteins is part of a larger superfamily of proteins that facilitate basic transport processes and are widely present in the various cells/tissue of human organism [1]. GLUT proteins are integral proteins of eukaryotic cell membranes. GLUT transporter isoforms differ in their kinetic characteristics, specificity for transported glucose, tissue localization, and regulatory mechanisms [2]. Some GLUT proteins, along with glucose, also transport other metabolites, such as galactose, water, and analgesics from the group of glycopeptides. GLUT proteins perform uniport, in which substances pass through the membrane in the direction of the concentration gradient. Thus, depending on the concentration, GLUT proteins transport metabolites into the cell or in the opposite direction. The entry of glucose into the cell, which occurs with the participation of GLUT proteins, often determines the viability of cells that are characterized by a high level of energy consumption. Nutrient metabolites, such as sugars, are transported through the blood vessels to the organs [[1], [2], [3]]. Endothelial cells (ECs) that line the walls of small vessels control nutrient metabolism. These ECs, especially those located in the area of the blood-brain barrier (BBB), contain many GLUT proteins [4]. The proper functioning of the brain is highly dependent on glucose, and its cells are especially sensitive to a decrease in its content. Transport of glucose into neurons occurs through the capillaries of the brain in several stages and with the participation of the GLUT-1 isoform [5]. This isoform is expressed in the membrane of ECs located at the border between the blood and intercellular space, as well as in the plasma membrane of astrocytes, whose function is important in the BBB [6]. The GLUT-1 proteins located in these places transport glucose from the blood to ECs, and from there to astrocytes. In them, glucose is converted into other sources of energy, which are transported to neurons [5,7]. Different tissues contain different isoforms of GLUT. For instance, in the cells of muscle and adipose tissue, glucose transport is carried out with the participation of GLUT-4. In the process of eating or after eating, glucose enters the cells of these tissues under the action of insulin. In this case, regulated transport of the GLUT-4 isoform to the cell surface occurs. This isoform is also called the insulin dependent transporter. The GLUT-4 protein is localized in intracellular vesicles that fuse with the plasma membrane. This ensures the delivery of the GLUT-4 transporter to the plasma membrane and increases the capacity of the transport process [8,9].

Activation of GLUT proteins, in particular GLUT-1, GLUT-2, GLUT-3 and GLUT-4, has been observed in gastric cancer, ovarian cancer, squamous cell carcinoma, glioblastomas, and meningiomas, and, in these tumors, where the Warburg effect is most pronounced, efforts have been made to develop new therapeutic strategies that include stopping the influx of glucose into the tumor cell [[10], [11], [12]]. This therapeutic intervention deserves more study, especially in intractable tumors with poor prognosis, such as glioblastoma, due to the fact that GLUT-1 contributes to the maintenance of the BBB. In particular, GLUT-1 and GLUT-3 have been identified as proteins that are activated in glioblastoma tissues, and the mechanism by which these glucose transporters are activated, in combination with a direct or indirect decrease in the amount of glucose these transporters allow into cells, may provide insight into the reversal of the Warburg effect observed in glioblastoma [13].

MicroRNAs (miRNAs) are small non-coding RNA molecules, approximately 18–22 nucleotides (nt) in length, that function as post-transcriptional regulators of gene expression in mammalian cells, which work through pair-coupling with complementary sequences in mRNA molecules, usually resulting in the downregulation of gene activity through translational repression [14]. MiRNAs are involved in most important cell biological processes such as control of cell cycle, cellular differentiation, proliferation, apoptosis, immune response, etc. Previous studies have indicated that miRNAs play a critical role in regulating oncogenesis of various human cancers through the inhibition or activation of GLUT proteins (Table 1) [[15], [16], [17], [18], [19], [20], [21], [22], [23], [24]]. However, to date, there have been few reports of miRNAs that can regulate GLUT expression in glioblastoma. Therefore, additional studies may lead to new approaches in antitumor therapy of glioblastoma and provide new insight into the epigenetic regulation of GLUT. Here we will focus on the potential regulatory role of a number of miRNAs on GLUT in glioblastoma.

**Table 1:** The role some miRNAs in human tumors by regulating GLUT expression and other gene targets.

miRNA	Tumor types	Expression	Study model	Gene-targets	GLUT types	Biological function	References
miR-340	Bladder cancer	Down	In vitro	PCNA, Bax and PI3K/AKT pathway	GLUT-1	Suppresses the proliferation and induces apoptosis in tumor cells	15
miR-150-5p	Oral squamous cell carcinoma	Up	In vitro and in vivo	PVT1	GLUT-1	Promotes cell proliferation, invasion, migration and inhibits tumor cells apoptosis	16
miR-21	Triple-negative breast cancer	Up	In vivo	Caspase-3	GLUT-1	Promote tumor progression	17
Let-7a-5p	Triple-negative breast cancer	Down	In vitro	N-cadherin and Vimentin	GLUT-12	Inhibits proliferation, migration and invasion	18
miR-1204	Ovarian squamous cell carcinoma	Up	In vitro	–	GLUT-1	Promote ovarian squamous cell carcinoma growth by increasing glucose uptake	19
miR-233-3p	Melanoma	Down	In vitro	IGF-1R/Akt axis	GLUT-1	Promote of melanoma cell glycolytic activity and progression	20
miR-143	Metastatic colorectal cancer	Down	In vitro	CPT1-A, IFN-γ, IL-2, Bcl-2, Bcl-6, and Eomes	GLUT-1	Anti-tumor effects of T cell by promoting memory T cell differentiation and metabolism reprogramming through GLUT-1	21
miR-455-5p	Hepatocellular carcinoma	Down	In vitro	IGF-1R/AKT axis	GLUT-1	Suppresses tumor cell growth and invasion	22
miR-210	Bladder cancer	Up	In vitro	HIF-1α	GLUT-1	High expression miR-210 may reflect hypoxia in bladder cancer.	23
miR-26b	Osteosarcoma	Down	In vitro	MMP-9, MMP-2, cyclin D1 and p27	GLUT-1	Inhibits proliferation, migration, invasion and apoptosis	24

**Abbreviations:** GLUT, glucose transporter; PCNA, proliferating cell nuclear antigen; Bax, Bcl-2-associated X protein; PI3K, phosphoinositide 3-kinases; PVT1, plasmacytoma variant translocation 1; IGF-1R, insulin-like growth factor type 1 receptor; CPT1-A, carnitine palmitoyltransferase 1A; IFN-γ, interferon‐gamma; IL-2, interleukin-2; Bcl-2, B-cell lymphoma 2**;** Bcl-6, B-cell lymphoma 6**;** HIF-1α, hypoxia-inducible factor 1-alpha**;** MMP-9, matrix metallopeptidase 9; MMP-2, matrix metallopeptidase 2.

## 2 GLUT in glioblastoma

It is known that in the central nervous system (CNS) GLUT-3 is a neuronal GLUT, and GLUT-1 is important for glucose uptake by astrocytes and glucose transport through the BBB [25]. In addition, GLUT-1 can also be ubiquitously expressed in all cells/tissues of the human body [26]. Differences in tissue expression between GLUT-1 and GLUT-3 may correlate with different energy requirements, where GLUT-3 compared to GLUT-1 has a five-fold higher affinity for glucose, allowing for better glucose uptake by cells in an environment with lower glucose concentrations [27]. Indeed, GLUT-3 activation in a subpopulation of less differentiated, highly metabolically plastic glioblastoma cells, termed brain tumor initiating cells (BTICs), promotes tumor cell survival under glucose restriction [28,29]. At the same time, BTICs are known to be more invasive than more differentiated glioblastoma cells [28]. GLUT-3 has also been upregulated in bevacizumab-resistant glioblastoma cells, but bevacizumab resistance is associated with a shift in metabolism and a more invasive and mesenchymal-like phenotype [30]. For example, Libby et al. have determined that GLUT-3 plays a role in mediating invasion of glioblastoma cells in addition to its role in metabolism, which is mediated by the C-terminus of the protein.

Among all existing GLUTs, GLUT-1 and GLUT-3 are the most activated in tumors, including glioblastoma. Their high degree of homology has made it incredibly interesting that GLUT-3 has such a significant correlation with patient prognosis (both overall survival and metastasis-free survival) when GLUT-1 is often not correlated [31]. The fact that GLUT-3 activation in various tumors, combined with a distinct invasive role, citing the work of Libby et al. a wider role of GLUT-3 in the invasion and metastasis of glioblastoma is assumed [29]. Invasion of glioblastoma into normal neural tissue eventually results in tumor recurrence very close to the margin of tumor resection, where this recurrence in almost all cases results in death. There is no “excess healthy tissue” in the CNS and therefore areas of healthy tissue at the border with the tumor cannot be resected to remove most of the invasive tumor. Thus, understanding the drivers of glioblastoma invasion will be critical to further drug development to improve patient outcomes for this formidable disease.

## 3 Hypoxia-regulated expression of GLUT in glioblastoma

**Fig. 1:**
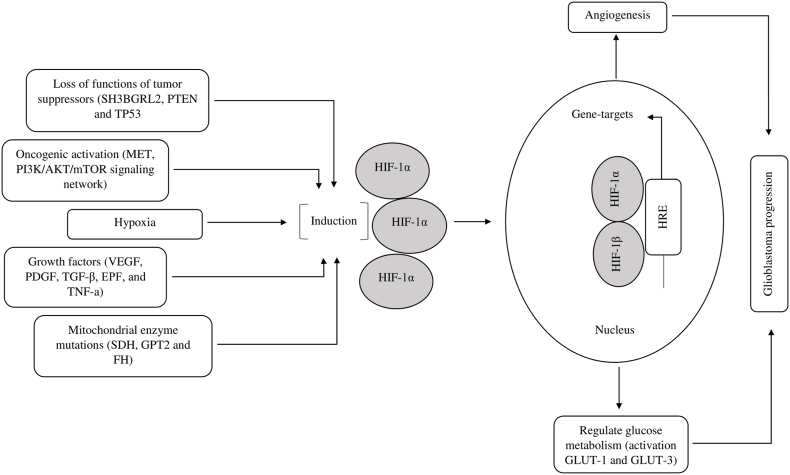
**Hypoxia-inducible factor 1α (HIF-1α) expression is deregulated in glioblastoma.** Overexpression of HIF-1α and activation of the HIF pathway in glioblastoma is caused by a combination of microenvironmental changes, such as changes in oxygen levels (hypoxia), increases in growth factors, and genetic abnormalities leading to loss of tumor suppressor function, oncogenic activation or deregulated mitochondrial function. Increased HIF-1α in glioblastoma cells translocate to the nucleus, binds to hypoxia-inducible factor 1 β (HIF-1β), recruits coactivators (e.g. p300/CBP) and activates the transcription of multiple genes involved in angiogenesis (e.g. vascular endothelial growth factor (VEGF)) and regulate glucose metabolism (e.g. glucose transporter 1 (GLUT-1) and glucose transporter 3 (GLUT-3) – thereby driving glioblastoma progression.

At all stages of glioblastoma oncogenesis, tumor cells are under hypoxic conditions, while hypoxic and anoxic areas are located heterogeneously in the tumor. Tumor cells and macrophages migrating to the foci of necrosis secrete factors that stimulate angiogenesis: vascular endothelial growth factor (VEGF), platelet-derived growth factor (PDGF), transforming growth factor β (TGF-β), epidermal growth factor (EPF), tumor necrosis factor-alpha (TNF-a), and activation of matrix metalloproteinases (MMPs) [32,33]. Angiogenic factors stimulate the proliferation and migration of ECs that form the tumor vasculature. At the same time, the ingrowing vessels have numerous defects: leaky post-capillary veins, weak blood flow due to the irregular course of the vessels, which poorly cope with providing the tumor with oxygen. If earlier it was believed that the main effect of hypoxia is the forced switching of the cell to a less favorable energy supply due to the impossibility of using a more profitable energy source - mitochondrial oxidation, now it is clear that hypoxia is a different state of the cell that supports and develops the tumor process [34]. Cells subjected to hypoxia show increased proliferation, arrest in differentiation by activating dedifferentiation genes (e.g. anti-CD4 antibody (Okt4) and Notch) increased ability to migrate and invade, resistance to apoptosis due to activation of anti-apoptotic genes of the B-cell lymphoma 2 (Bcl-2) family and stimulation of multidrug resistance [35]. There is a sharp increase in the synthesis of glycolysis enzymes: lactate dehydrogenase, glucose transporter and others, including GLUTs [36]. The main factor of hypoxia is the transcription factor hypoxia-inducible factor 1-alpha (HIF-1α), the content of which is regulated at the protein level: with a sufficient amount of oxygen, the O2 sensor is the enzyme prolyl oxidase, which oxidizes HIF-1α at prolines. HIF-1 is a heterodimeric protein consisting of two subunits of HIF-1α, regulated by oxygen concentration and HIF-1β, which is constantly expressed [37,38]. HIF-1α induces transcription of over 60 genes, including VEGF and GLUT, which are involved in biological processes such as angiogenesis and metabolic adaptation that promote and increase oxygen delivery to hypoxic areas (Fig. 1). Under hypoxic conditions, HIF-1α is not hydroxylated, remains stable and accumulates. Subsequently, HIF-1α and hypoxia-inducible factor 1- beta (HIF-1β) combine to form HIF-1 in the cell nucleus, activating (through influence on DNA): angiogenesis, indirectly through VEGF, erythropoiesis and glycolysis enzymes [[37], [38], [39], [40]].

Glioblastoma is the most common and most aggressive form of CNS tumor. The successive proliferation of tumor cells leads to a sharp increase in oxygen consumption, which causes hypoxia and an oxygen-free tumor microenvironment is created. It is known that HIF-1α plays an important role in the adaptation of the cellular environment to hypoxia by inducing the expression of various transcription factors in response to hypoxia, including GLUT [41]. In addition, it is known that hyperactivation of HIF-1α in the tumor leads to the ineffectiveness of radio- and chemotherapy and poor prognosis in glioblastoma. For example, Liu et al. showed that HIF-1α expression increases with increasing pathological grade of glioma [42]. Their results can also be interpreted as suggesting that oxygen deficiency may be related to the invasiveness of glioblastoma. The correlation between HIF-1α expression and pathological grade of gliomas suggests that HIF-1α expression should also be increased in response to oncogene activation and/or tumor suppressor gene inactivation during tumor progression. For example, it has been shown that p53 inactivation promotes an increase in HIF-1α expression, which ultimately leads to an increase in VEGF-mediated tumor angiogenesis [43]. At the same time, the authors also demonstrated that GLUT-3 was more detectable in grade 3-4 gliomas (World Health Organization (WHO) 2021) than in grade 1-2 gliomas (WHO 2021), indicating that GLUT-3 may be predominant glucose transporter in high-grade gliomas [42]. The results of their study show that the expression of HIF-1α and GLUT-3 is generally correlated, but with significant differences between them. There are certain unknown mechanisms, such as the regulatory relationship between HIF-1, GLUT-3 and miRNAs, that will be the focus of further research.

It is known that p21 has been identified as a widespread inhibitor of cyclin-dependent kinases, which mediates the arrest of the G1 phase, the p53-dependent cell cycle of a tumor cell, in response to various stress stimuli [44]. Jin et al. discovered a new function of p21 in glioblastoma, which is involved in the regulation of metabolism under hypoxic conditions [45]. The authors found that HIF-1α directly binds to the hypoxia response elements (HRE) of the p21 promoter to increase its transcriptional activity. In turn, p21 also promoted HIF-1α transcription at the mRNA level, retaining its function under conditions of oxygen deficiency. Thus, a positive correlation between p21 and HIF-1α enhances GLUT-1/lactate dehydrogenase A (LDHA) mediated glycolysis and exacerbates glioblastoma radioresistance.

## 4 MiRNA/HIF-1a axis in glioblastoma

As is known from the previous chapter, in many tumors, including glioblastoma, HIF-1α is deregulated with activation of a number of genes involved in angiogenesis (for example, VEGF), metabolic adaptation (for example, GLUT-1), cell survival (for example, the family Bcl-2) and in tumor metastasis (e.g., PDGF), stimulating tumor progression (Fig. 1). In addition to protein-coding genes, HIF-1α can activate the expression of numerous miRNAs under hypoxic conditions in glioblastoma. Huang et al. identified hypoxia-mediated downregulation of miR-224-3p as a novel inhibitor of hypoxia-induced autophagy in glioblastoma [46]. In the present study, the authors observed that HIF-1α expression increased while miR-224-3p expression decreased. In addition, inhibition of HIF-1α expression reduced the motility and chemosensitivity of tumor cells due to the negative regulation of miR-224-3p expression. Meanwhile, miR-224-3p has been found to inhibit autophagy by targeting autophagy-related gene 5 (ATG5). Together, the HIF-1α/miR-224-3p/ATG5 signaling pathway affects the motility and chemotherapy sensitivity of glioblastoma tumor cells by regulating hypoxia-induced autophagy. Moreover, miR-224-3p was one of the expressed miRNAs in glioblastoma under oxygen deficient conditions in some studies.

Xu et al. demonstrated that miR-20a expression is increased in grade 3-4 gliomas and relatively decreased in gliomas with the IDH1 R132H mutation [47]. The IDH1 R132H mutation increased the expression of HIF-1a, which led to a decrease in the expression of c-Myc and miR-20a. However, c-Myc activation led to the induction of miR-20a and was inactivated under hypoxic conditions. c-Myc activation also increased miR-20a expression in an *in vitro* model of glioblastoma with an IDH1 mutation. Taken together, these results suggest that HIF-1a is responsible for downregulating c-Myc expression, resulting in the observed decrease in miR-20a expression.

It is known that the suppression of HIF-1α has been investigated to sensitize glioblastoma cells to temozolomide (TMZ) treatment [48]. However, the underlying mechanism still remains elusive. Thus, Ge et al. demonstrated the potential role of miR-26a in glioblastoma to chemotherapy resistance [49]. They found a novel role of miR-26a to enhance TMZ resistance of glioblastoma cells *in vitro* and in vivo models. In addition, in their study was found that miR-26a was upregulated by HIF-1α as the pivotal molecular regulator of oxygen homeostasis under hypoxic conditions. In other study, Hu et al. presented that miR- 576-3p directly targets the 3′-untranslated region (3′-UTR) of HIF-1α mRNA in hypoxia-treated glioblastoma cells, where overexpression miR- 576-3p inhibits the migration and proangiogenic abilities of tumor cells [50].

**Fig. 2:**
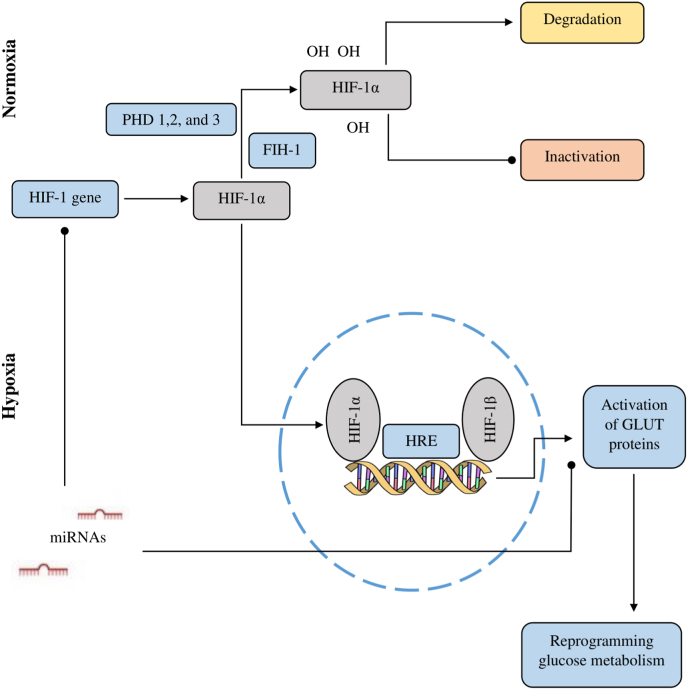
**Illustrative view of microRNAs (miRNAs) interference on hypoxia-inducible factor 1α (HIF-1α) pathway.** In normoxia, HIF-1a is subjected to degradation or inactivation, mediated by three prolyl hydroxylases (PHD1–3) and a single asparagine hydroxylase termed factor inhibiting HIF 1 (FIH-1), respectively. In hypoxic conditions, HIF-1α translocate to the nucleus and binds hypoxia-inducible factor 1β (HIF-1β), forming the transcriptional active hypoxia‐inducible factor (HIF) complex. The heterodimer recognizes the hypoxia-response element (HRE) and initiates transcription of gene-targets, such as glucose transporters (GLUTs). Some miRNAs, collectively called hypoxamiRs, target HIF-1α and GLUT mRNAs and inhibit HIF-1a and GLUT protein expression.

In summary, because HIF-1a plays a significant role in glucose metabolism with activation GLUT, the deregulation of these miRNAs might contribute to gliomagenesis (Fig. 2). However, how these miRNAs regulate GLUTs expression in glioblastoma under hypoxic conditions requires further study.

## 5 Analysis of experiments on the study of GLUT regulation by miRNAs

Aerobic glycolysis is a hallmark of glioblastoma, like any tumor. To date, great progress has been made in understanding the role of microRNAs in the regulation of glycolytic metabolism in glioblastomas. MiRNAs regulate glycolytic metabolism by controlling the expression of glycolytic proteins such as GLUT, which themselves are involved in the regulation of glycolysis. To date, several GLUT-regulating miRNAs are known to directly control the functioning of the components of the glycolytic pathway in other tumors [[15], [16], [17], [18], [19], [20], [21], [22], [23], [24]]. Moreover, other differentially expressed miRNAs in glioblastoma tissues and cells, which are not yet associated with the glycolytic metabolism of this tumor, play metabolic regulatory roles in other tumors. Although the differential expression of these miRNAs in glioblastoma may suggest a similar metabolic regulatory role in this tumor, functional verification studies are needed before such associations can be established.

Although the mechanism of the relational role for miR-183 on HIF-1a is not fully understood, Tanaka et al. provided evidence that miR-183 overexpression increases HIF-1a expression in glioblastoma cells [51]. In this study, the authors also observed significantly reduced levels of isocitrate dehydrogenase (NADP (+)) 2 (IDH2) and elevated levels of HIF-1a in all glioblastoma cell lines transfected with miR-183 mimic compared to tumor cell lines transfected with control RNA. In addition, using real-time polymerase chain reaction (real-time PCR), the authors demonstrated that VEGF and GLUT-1 mRNA transcription levels were increased in in vitro glioblastoma cells transfected with miR-183 mimiс compared to cells transfected with control RNA. These results indicate that miR-183 overexpression induces GLUT-1 activation in glioblastoma.

Recent studies have shown that GLUT-3 expression was increased in chemoresistant glioblastoma cells, where chemoresistance is associated with a shift in metabolism, as well as a more invasive and mesenchymal-like tumor phenotype [52,53]. Dai et al. demonstrated that upregulation of miR-106a suppresses GLUT-3 expression by targeting its 3′-UTR mRNA and results in tumor cell proliferation and inhibition of cellular glycolysis in glioblastoma cells [54]. In addition, a decrease in miP-106a expression and an increase in GLUT-3 levels indicate poor survival in patients with glioblastoma. These results suggest that GLUT-3 overexpression in glioblastoma is closely associated with an aggressive course and poor prognosis.

It is known that the phosphoinositide 3-kinase (PI3Ks)/Akt signaling pathway plays an important role in the regulation of glycolytic metabolism in glioblastoma [55]. Cheng et al. reported that long non-coding RNA (lncRNA) X-inactive specific transcript (lncRNA-XIST) is a tumor suppressor in glioblastoma by increasing miR-152 expression and, accordingly, suppressing lncRNA-XIST expression. leads to inhibition of proliferation, migration, and invasion of tumor cells with increased apoptosis [56]. Moreover, knockdown of lncRNA-XIST reduced the level of expression of GLUT-1 and GLUT-3, while this inhibition of lncRNA-XIST expression in glioblastoma cells is attenuated during hypoxia. The authors also showed that lncRNA-XIST functions as a ceRNA to regulate insulin receptor substrate 1 (IRS-1) by spongy miR-126 in glioblastoma cells. Taken together, this study demonstrated a novel molecular mechanism for the control of glycolysis, which was dependent on the lncRNA-XIST/miR-126/IRS1/PI3K/Akt signaling pathway, in altered glucose metabolism in glioblastoma. Guo et al. suggest that miR-451 is a novel tumor suppressor miRNA, where miR-451 can inhibit tumor cell proliferation and invasion by directly targeting GLUT-1 in glioblastoma cells [57]. The authors further investigated whether miR-451 could modulate glucose energy metabolism via the PI3K/Akt signaling pathway to downregulate GLUT-1 expression in glioblastoma. MiR-451 was also found to inhibit the PI3K/Akt signaling pathway. These data confirm that miR-451 is a key molecular switch that downregulates GLUT-1 expression, reduces glucose energy metabolism, and inhibits proliferation and invasion of glioblastoma cells in vivo and *in vitro*.

Specificity protein 1 (SP1), a member of a family of transcription factors like Kruppel factor and specificity protein (KLF/SP), is one of the first identified eukaryotic transactivators. Some studies report that SP1 expression is impaired in various types of tumors, including gliomas. Overexpression of SP1 plays an important role in the regulation of many vital oncoproteins such as VEGF. And SP1 overexpression is associated with poor clinical outcome [58,59]. Moreover, some studies have found a positive regulatory relationship between SP1 and GLUT-1, which themselves are also important regulators of glycolysis [60,61]. Yin et al. found that miR-181b can inhibit glucose metabolism (inactivates GLUT-1) and glioblastoma cell proliferation by targeting the 3′-UTR of SP1 mRNA [62]. Another study found that histone deacetylase 2 (HDAC2) knockdown was directly related to the regulation of metabolites in mitochondrial respiration and glycolysis through inhibition of GLUT-3 expression and ultimately led to glioblastoma cell death [63]. In addition, HDAC2 knockdown increased the expression of miP-3189, which was also implicated in the inhibition of GLUT-3 expression, leading to the inhibition of gliomagenesis and the formation of cancer stem cells (CSCs)-spheres, and inducing GSC death.

A number of studies report aberrant expression of miR-495 in several types of tumors, including glioblastoma [64,65]. For example, miR-495 is significantly downregulated in glioblastoma tissues and inhibits tumor cell proliferation by inhibiting cyclin-dependent kinase [65]. Nie et al. also demonstrated that miR-495 is significantly reduced in glioblastoma tissue [66]. In particular, their study provides direct evidence that downregulation of miP-495 in glioblastoma cells promotes cellular glycolysis through targeting GLUT-1. And all this naturally leads to the growth and development of the tumor.

Thus, understanding how glucose enters cells through the regulation of GLUT protein expression by miRNAs is one of the central issues for the design of effective therapeutic agents. Of course, more research will be needed to prove a link between the ability of miRNAs to regulate glucose metabolism and their ability to control the expression of GLUT proteins in glioblastoma. Table 2 presents studies that have studied the role of miRNAs in the regulation of GLUT protein expression in glioblastoma.

**Table 2:** Role of miRNAs in the regulation of GLUT proteins and other gene-targets expression in glioblastoma.

miRNAs	Expression	Study model	Gene-targets	GLUT types	Biological function	References
miR-183	Up	In vitro	IDH2, VEGF and HIF-1a	GLUT-1	Progress of gliomagenesis	51
miR-106a	Down	Bioinformatics analysis and *in vitro*	–	GLUT-3	Inhibits glioma cell glucose uptake and proliferation	54
miR‐126	Up	In vitro and in vivo	IRS1/PI3K/Akt pathway	GLUT-1 and GLUT-3	Promote tumor cell viability, migration, invasion, and resistance to apoptosis	56
miR-451	Down	In vitro and in vivo	LKB1/AMPK/PI3K/Akt	GLUT-1	Inhibits glioma cell proliferation and invasion	57
miR-181b	Down	In vitro and in vivo	SP1 and PKM2	GLUT-1	Suppresses glucose metabolism and cell proliferation	62
miR-3189	Down	In vitro and in vivo	HDAC2	GLUT-3	Inhibits glioblastoma tumorigenesis through regulating glucose metabolism and proliferation	63
miR-495	Down	In vitro	–	GLUT-1	Inhibit a metabolic shift in glioma cells.	66

**Abbreviations:** GLUT, glucose transporter; PI3K, phosphoinositide 3-kinases; HIF-1α, hypoxia-inducible factor 1-alpha**;** IDH2, isocitrate dehydrogenase; VEGF, vascular endothelial growth factor; IRS1, insulin receptor substrate 1; LKB1, liver kinase B1; AMPK, AMP-activated protein kinase; SP1, specificity protein 1; PKM2, glycolytic enzyme pyruvate kinase M2; HDAC2, histone deacetylase 2.

## 6 Conclusion and final remarks

Glioblastoma cells show enhanced glucose metabolism compared to normal tissue. The resulting significant increase in glucose requirements suggests a corresponding increase in glucose transport across the plasma membrane. Most tumor types overexpress members of the GLUT family that are present in their respective tissue of origin in non-cancerous conditions. Moreover, due to the energy requirement to fuel uncontrolled proliferation, tumor cells often express GLUTs that are not normally present in these tissues. In human studies, GLUT-1 and GLUT-3 overexpression has been correlated with a number of characteristics of glioblastoma, including increased invasive potential, proliferative activity, and decreased patient survival. It has been shown that GLUT-1 and GLUT-3 expression correlates with the level of tumor hypoxia. Hypoxia has been shown to increase GLUT-1 and GLUT-3 gene expression. It is known that the presence of hypoxia in glioblastoma leads to its resistance to radiation and chemotherapy and is associated with a more aggressive phenotype. MiRNAs are involved in the regulation of various biological processes. Biochemically, miRNAs also regulate glucose metabolism, either directly by acting on key enzymes in metabolic pathways, or indirectly by modulating the expression of important transcription factors like GLUT. A number of studies demonstrated in this work have shown that altered metabolic pathways with GLUT activation in glioblastoma are tightly regulated by miRNAs.

In conclusion, our review provides the current status of understanding regulating GLUT expression in glioblastoma by miRNAs that would provide benefits for research guidance in this emerging field the future. For a more comprehensive review of micro- and other non-coding RNA changes associated with glioblastoma, see recent reviews by Gareev et al. (2022) and Beylerli et al. (2022) [67,68].

## Funding

None.

## Declaration of competing interest

The authors declare no conflict of interest, financial or otherwise.
